# Effectiveness of a multi-component educational intervention on perceived self-efficacy and occupational well-being among health workers: a randomized controlled trial in Southwest Iran

**DOI:** 10.1186/s12913-026-15084-y

**Published:** 2026-07-06

**Authors:** Hojatallah Nadeali, Zohreh Karimiankakolaki

**Affiliations:** 1 Department of Health, Islamic Azad University, Shk.C., Shahrekord, Iran; 2 Social Determinants of Health Research Center, Shk.C., Islamic Azad University, Shahrekord, Iran

**Keywords:** Health literacy, Perceived self-efficacy, Occupational well-being, Health workers

## Abstract

**Background:**

Health literacy is a critical social determinant of health (SDH) that can influence the professional performance of healthcare workers. However, evidence from randomized controlled trials (RCTs) on the effectiveness of structured health literacy training on self-efficacy and occupational well-being among primary health workers is limited. This study aimed to evaluate the effectiveness of a multi-component educational intervention (integrating health literacy fundamentals, self-efficacy theory, communication skills, and stress management) on perceived self-efficacy and occupational well-being.

**Methods:**

This RCT employed a pretest-posttest design with a control group. A total of 88 health workers were randomly allocated to either an intervention group (*n* = 44) or a control group (*n* = 44) using two-stage random sampling. The intervention group received an 8-session health literacy training program (60 min per session, two sessions per week), while the control group received no training. Data were collected using the Ansari Self-Efficacy Questionnaire (20 items, three dimensions: perseverance, self-regulation, effort) and the Zheng Occupational Well-being Questionnaire (18 items, three dimensions: personal life well-being, work well-being, psychological well-being). Data were analyzed using repeated measures ANOVA and MANCOVA in SPSS-26.

**Results:**

No significant differences were found between the two groups at baseline (*p* > 0.05). MANCOVA results showed a significant effect of the intervention on the combined dependent variables (self-efficacy and occupational well-being) (*P* < 0.001, η² = 0.247, power = 0.998). In the intervention group, mean self-efficacy scores increased from 75.40 to 82.00 (*P* = 0.001), and mean occupational well-being scores increased from 87.75 to 91.72 (*P* = 0.004), while no significant changes were observed in the control group. Additionally, a strong positive correlation was found between self-efficacy and occupational well-being in the post-test phase (*r* = 0.518, *P* < 0.001).

**Conclusion:**

A structured multi-component educational intervention significantly improves perceived self-efficacy and occupational well-being among health workers. Based on these findings, such training may be considered for integration into in-service education programs, particularly in similar primary healthcare settings. However, further research in larger and more diverse samples is needed before widespread policy recommendations can be made.

**Clinical trial number:**

Not applicable.

## Introduction

Effective healthcare delivery depends on a competent and psychologically healthy workforce [1]. According to the World Health Organization, social determinants of health are the conditions in which people are born, grow, live, work, and age [1]. The Commission on Social Determinants of Health has recommended health literacy as a major determinant of health status [2]. Health is recognized as an individual and social value and one of the most fundamental human rights [3].

Health literacy (HL) refers to an individual’s ability to obtain and use knowledge and information to maintain and improve health in a manner appropriate to the individual’s and system’s conditions [4]. Research has shown that low health literacy leads to adverse health effects, including increased risk in emergencies, lack of self-confidence and social competence, chronic diseases, more hospitalizations, greater medication use, less ability to take medications appropriately, difficulties in interpreting medication instructions, and poor reporting of one’s health status [5].

Health workers, as the first point of contact in the healthcare system, play a vital role in health education, disease prevention, and primary care provision [1]. Additionally, low health literacy can lead to adverse outcomes such as difficulties in interpreting medication instructions and poor reporting of health status [6]. The results of Moeini and colleagues’ study showed that, given the importance of social, economic, and environmental factors in individuals’ health, more attention to the health literacy of employees is essential [7].

The concept of self-efficacy is the ability to perform a specific activity and the expectation of having the ability to successfully perform a specific behavior [8]. Educational factors play an important role in the development and growth of self-efficacy. The structure of self-efficacy can be used as a theoretical basis in many health education programs to create and promote health behaviors [9]. Individuals with high self-efficacy believe that they are able to effectively control important life events [10]. Bandura defines self-efficacy as an individual’s judgment about their abilities to perform specific actions. Perceived self-efficacy is an important component of an individual’s performance because it acts as an independent part of their basic skills [11].

Occupational well-being is a multidimensional construct derived from the field of positive occupational health psychology [12]. Horn and colleagues define occupational well-being as a positive evaluation that an individual makes of various aspects of their job, including emotional, motivational, behavioral, cognitive, and psychosomatic dimensions [13]. In the study by Shafiei and Nasiri, both health literacy and well-being had a direct impact on quality of life and were strong predictors of it, and the two variables of health literacy and psychological well-being had a significant relationship with each other [14]. Fiedler and colleagues in a study on German industrial managers showed a significant relationship between health literacy and well-being in managers [15].

Despite this evidence, there is a gap in the literature regarding interventional studies examining the causal effect of health literacy training on self-efficacy and occupational well-being among health workers in Iran. A recent study by Shafiey and Karimiankakolaki (2025) demonstrated that a structured communication skills training program significantly enhanced both self-efficacy and communication abilities among primary healthcare workers in southwest Iran, suggesting that targeted educational interventions can improve healthcare workers’ psychological resources [16].

The theoretical mechanism underlying this study is Bandura’s social cognitive theory, which posits that self-efficacy is enhanced through mastery experiences, vicarious learning, verbal persuasion, and emotional arousal management. A structured multi-component educational intervention significantly improves perceived self-efficacy and occupational well-being among health workers compared to a control group.

However, no study has specifically examined the effectiveness of a health literacy intervention on both self-efficacy and occupational well-being simultaneously among health workers in the Iranian primary healthcare system. Therefore, this study was designed to answer the following question: Does a structured multi-component educational intervention significantly improve perceived self-efficacy and occupational well-being among health workers employed in the Saman healthcare network (Chaharmahal and Bakhtiari Province, southwest Iran) compared to a control group?

## Methods

### Study design

**Fig. 1:**
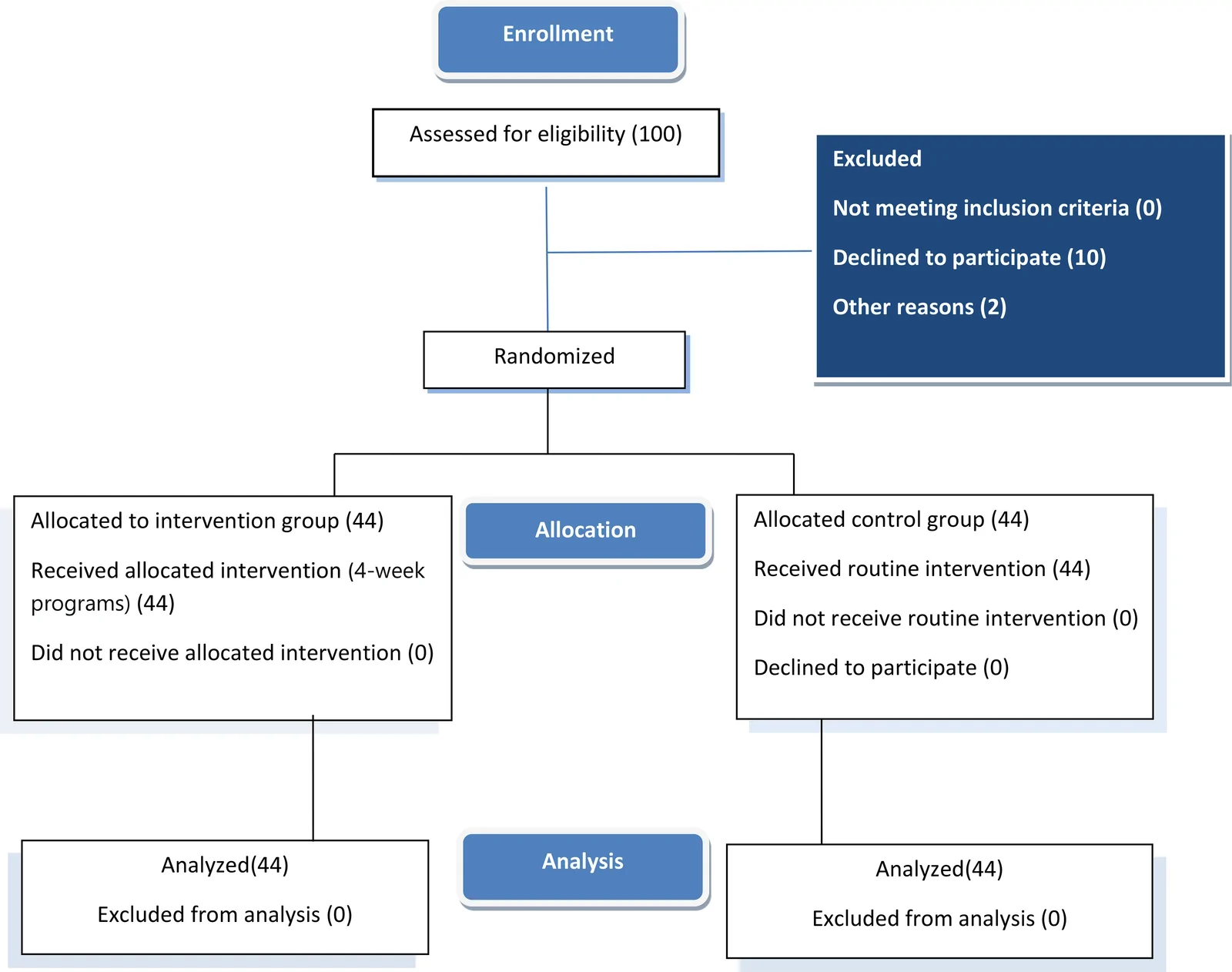
Research steps

This was a randomized controlled trial (RCT) with a pretest-posttest design and a control group. The post-test was conducted immediately after the last intervention session. The study was conducted between March and September 2025 in Saman city, Chaharmahal and Bakhtiari Province, Iran. The study protocol was developed according to the CONSORT guidelines. The CONSORT flow diagram illustrates participant progression through the trial. The study flow is presented in Fig. 1.

### Study setting

This study was conducted in Saman city, located in Chaharmahal and Bakhtiari Province, southwest Iran. Saman has a mixed urban-rural population. Primary healthcare in this area is delivered through a network of health houses, rural health centers, and an urban health center, following the Iranian PHC (Primary Healthcare) system.

### Participants and Setting

The study population included all health workers employed in the Saman Healthcare Network.

- **Inclusion criteria**: Full-time employment, minimum associate degree (post-diploma) education, willingness to participate.
- **Exclusion criteria**: Absence from more than two training sessions (for the intervention group), incomplete questionnaires, job resignation or transfer during the study.

### Sample size and randomization

Sample size was calculated using the standard formula (α = 0.05, power = 0.80, effect size d = 0.5, attrition rate = 10%), resulting in 44 participants per group (total *N* = 88). Four health centers were randomly selected. Then, from eligible workers within these centers, 88 individuals were randomly allocated (using a random number table) into intervention (*n* = 44) and control (*n* = 44) groups.

### Educational intervention

The intervention consisted of 8 sessions (60 min each) over 4 weeks (two sessions per week). The content was based on the validated protocol by Fischer, Barkley, Smallish, and Fletcher (2005), adapted for Iranian health workers. The control group received no training (waiting list). Table 1 summarizes the session content.

**Table 1:** Content of training sessions

Session	Title	Key Content
1	Fundamentals of Health Literacy	WHO definition, four core skills (access, understand, appraise, apply), group discussion
2	Self-Efficacy (Bandura’s Theory)	Four sources of self-efficacy, positive self-talk exercise
3	Occupational Well-being & Work-Life Balance	Dimensions of well-being, signs of burnout, box scheduling technique
4	Effective Communication Skills	Active listening, assertiveness, constructive feedback, role-playing
5	Stress Management & Emotional Regulation	Workplace stressors, ABC cognitive restructuring, mindfulness breathing
6	Evidence-Based Problem Solving	7-step problem-solving model, searching credible health information
7	Practical Review & Peer Modeling	Review of key points, experience panel, group discussion
8	Personal Action Plan	Developing a 3-month action plan, commitment to training colleagues

### Data collection instruments

Data were collected at two time points: pre-test (before the intervention) and post-test (immediately after the last intervention session) using:

A. **Demographic Questionnaire**: Age, gender, marital status, education level, work experience.
B. **Ansari Self-Efficacy Questionnaire (2010)**: A 20-item scale with three subscales: perseverance (7 items), self-regulation (7 items), and effort (6 items). Items are rated on a 5-point Likert scale (1 = strongly disagree to 5 = strongly agree). Total scores range from 20 to 100. In the present study, Cronbach’s alpha was 0.89 for the total scale [17].
C. **Zheng Occupational Well-being Questionnaire (2015)**: An 18-item scale with three subscales: personal life well-being (6 items), work well-being (6 items), and psychological well-being (6 items). Items are rated on a 7-point Likert scale (1 = strongly disagree to 7 = strongly agree). Total scores range from 18 to 126. In this study, Cronbach’s alpha was 0.82 for the total scale [18].

### Ethical considerations

Ethical approval was obtained from the Ethics Committee of Islamic Azad University, Shahrekord Branch (code: IR.IAU.SHK.REC.1404.077). All participants provided written informed consent.

### Statistical analysis

Data were analyzed using SPSS version 26. Normality was checked with the Kolmogorov-Smirnov test, and homogeneity of variances with Levene’s test. Chi-square and independent t-tests were used for baseline comparisons. Between-group differences at post-test were assessed using ANCOVA (controlling for pre-test scores) for each outcome, and MANCOVA for combined dependent variables. Within-group changes from pre-test to post-test were assessed using repeated measures ANOVA for each group separately. Pearson correlation was used to examine relationships between variables. A P-value < 0.05 was considered statistically significant.

## Results

A total of 88 participants completed the study with zero attrition (no participant missed more than two sessions or submitted incomplete questionnaires). All 44 participants in the intervention group attended at least 6 out of 8 sessions, and all questionnaires were fully completed. The majority were female (61.4%). No significant differences were found between the two groups at baseline in terms of age, gender, education, marital status, or work experience (*P* > 0.05), indicating successful randomization (Table 2).

**Table 2:** Baseline characteristics of participants by study group

Variable	Intervention Group (*n* = 44)	Control Group (*n* = 44)	*P*-value*
Gender (Female), n (%)	26 (59.1%)	28 (63.6%)	0.663
Marital status (Married), n (%)	31 (70.5%)	36 (81.8%)	0.210
Education (Bachelor & above), n (%)	29 (65.9%)	27 (61.4%)	0.658
Work experience (11–20 years), n (%)	24 (54.5%)	23 (52.3%)	0.832
Age (Mean ± SD)	31.9 ± 7.50	33.2 ± 8.10	0.430

*Analyzed by Chi-square (categorical) and independent t-test (continuous)

To examine changes over time within the intervention group, repeated measures ANOVA was conducted comparing pre-test to post-test scores. To compare the two groups at post-test while controlling for pre-test scores, ANCOVA was performed for each outcome. As presented in Table 3, the intervention group showed significant improvements from pre-test to post-test in total self-efficacy (F(1,43) = 24.56, *p* < 0.001, partial η²=0.363) and all its subscales, as well as in total occupational well-being (F(1,43) = 9.82, *p* = 0.004, partial η²=0.186) and all its subscales. No significant within-group changes were observed in the control group for any outcome (*p* > 0.05 for all).

Furthermore, ANCOVA results revealed that the intervention group scored significantly higher than the control group at post-test on all outcomes (all *p* < 0.05). A MANCOVA was then performed on the combined dependent variables (self-efficacy and occupational well-being). As shown in Table 4, the MANCOVA revealed a significant overall effect of the intervention (Pillai’s Trace = 0.247, F(2,83) = 13.62, *p* < 0.001, partial η²=0.247, power = 0.998).

**Table 3:** Within-group repeated measures ANOVA results (intervention group) and between-group ANCOVA results for self-efficacy and occupational well-being

Variable	Phase	Intervention Group Mean (SD)	Control Group Mean (SD)	*P*-value*	F (1,43)	*p*-value	Partial η²
Self-efficacy (Total)	Pre-test	75.40 (8.52)	73.13 (7.89)	**0.001**	24.56	< 0.001	0.363
	Post-test	82.00 (6.81)	74.81 (5.39)				
Perseverance	Pre-test	27.00 (3.92)	25.97 (3.88)	**0.001**	18.22	< 0.001	0.298
	Post-test	29.38 (3.02)	26.61 (2.90)				
Self-regulation	Pre-test	26.22 (3.38)	25.52 (3.06)	**0.001**	20.15	< 0.001	0.319
	Post-test	28.52 (2.59)	26.31 (2.24)				
Effort	Pre-test	22.18 (3.03)	21.63 (3.77)	**0.001**	15.87	< 0.001	0.270
	Post-test	24.09 (2.79)	21.88 (2.37)				
Occupational WB (Total)	Pre-test	87.75 (14.41)	85.00 (9.03)	**0.004**	9.82	0.004	0.186
	Post-test	91.72 (12.02)	84.56 (8.79)				
Personal life WB	Pre-test	29.29 (4.98)	28.72 (3.32)	**0.036**	4.68	0.036	0.098
	Post-test	30.25 (4.07)	28.56 (2.99)				
Work WB	Pre-test	28.27 (5.30)	27.06 (4.61)	**0.003**	9.92	0.003	0.187
	Post-test	29.95 (4.63)	26.77 (4.57)				
Psychological WB	Pre-test	30.18 (5.30)	29.20 (3.70)	**0.014**	6.44	0.014	0.130
	Post-test	31.52 (4.64)	29.22 (3.78)				

WB = Well-being. SD = Standard Deviation. The P-value* column shows between-group ANCOVA results at post-test (controlling for pre-test scores) with df = 1,85. The F (1,43), p-value, and Partial η² columns show within-group repeated measures ANOVA results for the intervention group only (comparing pre-test to post-test). For the control group, no significant within-group changes were observed (*p* > 0.05 for all outcomes)

**Table 4:** MANCOVA results for the combined effect of the intervention on dependent variables at post-test

Analysis	Effect	F	df1	df2	*p*-value	Partial η²	Power
MANCOVA	Combined dependent variables (self-efficacy & occupational well-being)	13.62	2	83	< 0.001	0.247	0.998

MANCOVA was performed controlling for pre-test scores. Pillai’s Trace is reported. The significant MANCOVA was followed by univariate ANCOVAs (presented in Table 3)

Pearson correlation analysis revealed a strong positive correlation between total self-efficacy and total occupational well-being at the post-test phase (*r* = 0.518, *P* < 0.001). All dimensions of self-efficacy were positively correlated with all dimensions of occupational well-being (Table 5).

**Table 5:** Pearson correlation matrix between self-efficacy and occupational well-being at post-test

Variable	1	2	3	4	5	6	7	8
1. Self-efficacy total	1							
2. Perseverance	0.803**	1						
3. Self-regulation	0.775**	0.435**	1					
4. Effort	0.761**	0.379**	0.426**	1				
5. Occupational WB total	0.518**	0.454**	0.370**	0.380**	1			
6. Personal life WB	0.452**	0.373**	0.363**	0.320**	0.854**	1		
7. Work WB	0.421**	0.385**	0.310**	0.283**	0.890**	0.640**	1	
8. Psychological WB	0.486**	0.431**	0.301**	0.395**	0.874**	0.627**	0.663**	1

**Correlation is significant at the 0.01 level (2-tailed). WB = Well-being

Footnote: High correlations between self-efficacy subscales (*r* > 0.75) suggest some construct overlap, which is expected given that they measure related facets of the same broader construct

## Discussion

This randomized controlled trial demonstrated that a structured health literacy educational intervention significantly improved both perceived self-efficacy and occupational well-being among health workers. The intervention group showed significant increases in self-efficacy (from 75.40 to 82.00, *P* = 0.001) and occupational well-being (from 87.75 to 91.72, *P* = 0.004), while the control group showed no significant changes. A strong positive correlation was found between self-efficacy and occupational well-being (*r* = 0.518, *P* < 0.001). These findings are consistent with the broader literature on educational interventions in healthcare settings [7, 17, 16].

### Effect on self-efficacy

The significant improvement in self-efficacy can be explained by Bandura’s social cognitive theory [10, 11]. According to Bandura, perceived self-efficacy plays a crucial role in cognitive development and functioning, as individuals with higher self-efficacy are more likely to approach challenging tasks as opportunities for mastery rather than as threats to be avoided [11]. Our intervention activated all four sources of self-efficacy as outlined by Bandura: (1) mastery experiences through role-playing and practical exercises, (2) vicarious experiences by observing successful peers, (3) verbal persuasion through facilitator feedback, and (4) management of emotional states via stress reduction techniques. As noted by Shafiey and Karimiankakolaki (2025), educational interventions that incorporate active learning strategies such as role-playing and group discussions are particularly effective in enhancing self-efficacy because they provide participants with direct mastery experiences and vicarious learning opportunities [16].

Our findings align with previous studies. Motamedi and colleagues showed that self-efficacy-based educational interventions improved health literacy and self-efficacy in adolescents [19]. Mojadam and colleagues found a significant positive correlation between health literacy and self-efficacy in hypertensive patients [20]. Similarly, Karimi and colleagues reported the same relationship in patients with type 2 diabetes [21]. While these were cross-sectional, our RCT provides causal evidence.

Khandan and colleagues demonstrated that self-care training based on Orem’s theory increased health literacy in cancer patients [23]. Ghodsizadeh and colleagues found a significant relationship between breastfeeding self-efficacy and health literacy in mothers [22]. Our study extends these findings by showing the same effect in health workers.

### Effect on occupational well-being

The improvement in occupational well-being can be attributed to several mechanisms. First, enhanced health literacy reduces role ambiguity by improving understanding of complex protocols. Second, problem-solving skills increase perceived control over work situations. Third, improved communication skills enhance social support from colleagues. Fourth, stress management techniques help regulate emotional responses to workplace stressors. Shafiey and Karimiankakolaki (2025) similarly reported that communication skills training significantly improved both self-efficacy and communication abilities, suggesting that these constructs are interdependent and mutually reinforcing [16].

Our findings are consistent with Ehsanifarid and colleagues, who found a significant relationship between health literacy and occupational well-being in university employees [24]. Tang and colleagues reported a strong positive correlation between mental health literacy and workplace well-being among Chinese civil servants, with regulatory emotional self-efficacy playing a mediating role [27]. Peng and colleagues demonstrated that occupational self-efficacy partially mediated the relationship between sleep quality and occupational well-being [25].

### Correlation between self-efficacy and occupational well-being

The strong positive correlation between self-efficacy and occupational well-being is consistent with the broader literature. Singh and colleagues found a positive relationship between self-efficacy and workplace well-being among Indian manufacturing managers [28]. In Iran, Abkhiz and Michaeli Manee showed that cognitive self-regulation affects occupational well-being through reducing burnout and increasing job satisfaction [29]. Jahanbazi and Lotfizadeh demonstrated a significant correlation between professional behavior and occupational well-being among nurses [30]. Additionally, Shafiei and Nasiri (2020) found that health literacy and psychological well-being had a significant relationship with each other in patients with type 2 diabetes [14].

This correlation likely reflects a virtuous cycle: higher self-efficacy leads to better task performance and positive feedback, which increases well-being; higher well-being, in turn, provides emotional resources to maintain and further develop self-efficacy [26, 31]. Basiri and colleagues previously reported that educational interventions improve both self-learning and communication skills among health workers, supporting the notion that training programs can simultaneously enhance multiple psychological resources [16]. Furthermore, Shafiey and Karimiankakolaki (2025) emphasized that healthcare workers with higher self-efficacy demonstrate greater competence, intrinsic motivation, and adaptability when facing challenging situations, which directly contributes to their overall occupational well-being [16].

### Strengths and limitations

#### Strengths

This study has several strengths, including the rigorous RCT design with a control group, a theory-based intervention grounded in Bandura’s framework, use of validated instruments with good reliability (Cronbach’s alpha > 0.82), assessment of multiple outcomes, a blended learning approach combining workshops with active strategies, and a 0% attrition rate.

#### Limitations

Several limitations should be acknowledged. First, the absence of a long-term follow-up limits our ability to assess the sustainability of the intervention effects over time. Second, reliance on self-report measures may introduce social desirability bias. Third, blinding of participants and outcome assessors was not possible due to the nature of the educational intervention, which may have introduced performance and detection bias (Hawthorne effect). However, the strong positive correlation between self-efficacy and occupational well-being (*r* = 0.518) supports the genuine effect of the intervention rather than mere expectancy effects. Fourth, the study was conducted in a single city, limiting generalizability. Fifth, the sample was predominantly female and well-educated. Sixth, no objective performance measures were included. Seventh, the study was not registered in a clinical trials registry; this is acknowledged as a limitation.

### Implications and future research

#### Practical implications

Healthcare managers should consider integrating health literacy training into routine in-service education programs. The intervention was low-cost (8 sessions, one facilitator) but produced significant improvements. Health policymakers should recognize health literacy as an important workforce development issue.

#### Future research

Studies with longer follow-up periods (6–12 months), larger and more diverse samples, inclusion of objective performance measures, cost-effectiveness analyses, and mixed-methods approaches are recommended.

## Conclusion

This randomized controlled trial provides robust evidence that a structured multi-component educational intervention significantly improves both perceived self-efficacy and occupational well-being among primary health workers in Iran. The intervention group showed meaningful improvements in both outcomes compared to the control group, and a strong positive correlation was found between self-efficacy and occupational well-being, indicating that these two constructs are closely interrelated.

Based on these findings, multi-component educational training may be considered for integration into in-service education programs, particularly in similar Iranian PHC settings. However, the findings need replication in larger, more diverse samples (including less educated workers, rural areas, and different cultural contexts) before widespread policy recommendations can be made.

## Data Availability

No datasets were generated or analysed during the current study.
